# Advancements and Challenges in Antiamyloid Therapy for Alzheimer's Disease: A Comprehensive Review

**DOI:** 10.1155/2024/2052142

**Published:** 2024-07-23

**Authors:** Semira Abdi Beshir, Nadia Hussain, Vineetha Bharathan Menon, Amal H. I. Al Haddad, Rahaf Adnan Kh. Al Zeer, Asim Ahmed Elnour

**Affiliations:** ^1^ Department of Pharmacy Practice Dubai Pharmacy College for Girls, Dubai, UAE; ^2^ Department of Pharmaceutical Sciences College of Pharmacy Al Ain University, Al Ain, UAE; ^3^ AAU Health and Biomedical Research Centre Al Ain University, Abu Dhabi, UAE; ^4^ Department of Pharmacy Practice College of Pharmacy Gulf Medical University, Ajman, UAE; ^5^ Chief Operations Office Sheikh Shakhbout Medical City (SSMC) PureHealth, Abu Dhabi, UAE; ^6^ Dubai Pharmacy College for Girls, Dubai, UAE; ^7^ College of Pharmacy Al Ain University Abu Dhabi Campus, Abu Dhabi, UAE

## Abstract

Alzheimer's disease (AD) is a progressive neurodegenerative disorder caused by the accumulation of amyloid-beta (A*β*) proteins and neurofibrillary tangles in the brain. There have been recent advancements in antiamyloid therapy for AD. This narrative review explores the recent advancements and challenges in antiamyloid therapy. In addition, a summary of evidence from antiamyloid therapy trials is presented with a focus on lecanemab. Lecanemab is the most recently approved monoclonal antibody that targets A*β* protofibrils for the treatment of patients with early AD and mild cognitive impairment (MCI). Lecanemab was the first drug shown to slow cognitive decline in patients with MCI or early onset AD dementia when administered as an infusion once every two weeks. In the Clarity AD trial, lecanemab was associated with infusion-site reactions (26.4%) and amyloid-related imaging abnormalities (12.6%). The clinical relevance and long-term side effects of lecanemab require further longitudinal observation. However, several challenges must be addressed before the drug can be routinely used in clinical practice. The drug's route of administration, need for imaging and genetic testing, affordability, accessibility, infrastructure, and potential for serious side effects are some of these challenges. Lecanemab's approval has fueled interest in the potential of other antiamyloid therapies, such as donanemab. Future research must focus on developing strategies to prevent AD; identify easy-to-use validated plasma-based assays; and discover newer user-friendly, and cost-effective drugs that target multiple pathways in AD pathology.

## 1. Introduction

Currently, over 55 million people worldwide have dementia, with more than 60% of them living in low- and middle-income countries. Each year, almost 10 million new cases of dementia are reported. Alzheimer's disease accounts for approximately 60-70% of all cases [1]. In the United States, currently, 6.9 million individuals currently suffer from Alzheimer's dementia, and the prevalence is increasing alongside the growth in the geriatric population [2]. AD is characterized by complex, progressive, chronic neuronal dysfunction that diminishes the quality of life and the ability to perform activities of daily living, including speaking, reasoning, attentiveness, judgment-making, or memory [3]. Additionally, it is associated with behavioral changes and psychiatric features [4].

Several factors contribute to AD pathogenesis. AD pathogenesis's hallmarks are beta-amyloid (A*β*) plaques [5] and neurofibrillary tangles (NFTs), which interrupt neuronal communication. Additionally, axonopathy, oxidative stress, and transport impairment have been linked to the development of AD [6]. Management of AD focuses on symptom management and the underlying pathology of the disease. Older drugs are mainly used to control the symptoms associated with AD and have no disease-modifying effects. However, in the past two decades, the focus has shifted to targeting the beta-amyloid (A*β*) protein. More extensive studies on drugs targeting A*β* protein are underway, particularly after the accelerated approval of the first disease-modifying therapy, aducanumab, for patients with mild AD and mild cognitive impairment (MCI) [7]. Lecanemab has now been approved for the same indication, two years after the approval of aducanumab. In addition to lecanemab and aducanumab, donanemab is being studied in a phase III (TRAILBLAZER-ALZ 3) trial [NCT05026866], which is expected to be completed by 2027. In this review, we aim to summarize the evidence from lecanemab clinical trials, compare it to other antiamyloid therapies, discuss challenges for the routine use of lecanemab therapy, and explore the impact of drug approval on AD patients, caregivers, and the general population. In addition, emerging AD therapies are summarized.

### 1.1. Pathogenesis of AD

AD is a neurodegenerative disorder involving cortical atrophy, resulting in a progressive decline in cognitive function. Advanced age increases the risk of AD, potentially owing to accumulating oxidative damage [8], vascular changes, inflammation, or cellular exhaustion. A*β* accumulation and tau tangles are the two most common proteins implicated in AD pathogenesis [9, 10]. A*β* pathology contributes to tau dysfunction. Deposition of A*β* and tau proteins consequently results in oxidative stress, leading to neuronal inflammation [11]. This inflammation worsens AD, with microglia identified as a contributing factor. In addition, lower levels of acetylcholine (ACh) [12], *β*-secretase [13], and *γ*-secretase in the brain contribute to AD pathogenesis. Furthermore, infectious agents such as HIV have also been linked to AD pathogenesis [14]. As AD is a heritable disorder, genetic risk factors, particularly ApoE alleles, contribute to AD [15, 16]. Other genetic variants associated with AD include ApoE, TREM2, CR1, CD33, CLU, BIN1, CD2AP, PILRA, SCIMP, PICALM, SORL1, SPI1, and RIN3 [17]. Recently, the role of cholesterol in the pathogenesis of AD has been investigated, particularly because of the link between the apolipoprotein E type 4 allele (ApoE4), a cholesterol cotransporter, and AD [18]. Dysfunction in cholesterol metabolism has been implicated in white matter injury and worsening of AD [19]. Further research into the molecular mechanisms, diagnostics, and treatment is ongoing to better understand and combat AD.

### 1.2. Antiamyloid Beta (A*β*) Therapies for AD

Trials of drugs that target A*β* have failed over several decades, either due to a lack of effectiveness or associated side effects [16–18]. Anti-A*β* therapies (e.g., bapineuzumab, solanezumab, crenezumab, aducanumab, lecanemab, gantenerumab, and donanemab) have been developed and investigated to reduce A*β* protein in the brain and halt the progression of AD. Phase III trials of bapineuzumab and solanezumab did not meet their primary endpoints and were discontinued [16–18]. Poor blood-brain barrier penetration of earlier antiamyloid therapies has been implicated as the cause of the lack of an effective delivery system [20]. However, crenezumab, gantenerumab, and donanemab are still under investigation [21]. Recent antiamyloid therapies (aducanumab, lecanemab, and donanemab) have successfully reduced A*β* plaques in the brain. Though these latter three antiamyloid therapies have been shown to effectively reduce A*β*, only lecanemab and donanemab were shown to reduce cognitive decline [22–24]. The mixed results obtained from trials targeting the A*β* protein have led to skepticism regarding the amyloid hypothesis as the main underlying cause of AD.

### 1.3. Comparison of Antiamyloid Therapies

Antiamyloid therapies differ in terms of their binding profiles, effectiveness in removing amyloid protein from the brain, frequency of administration, effect on halting cognitive decline, and potential side effects. A study that evaluated the binding affinity of lecanemab, aducanumab, and gantenerumab showed that all of these therapies exhibited low affinity to monomers [25]. Gantenerumab shows better binding to monomers compared to the other two. However, lecanemab has superior binding to protofibrils compared to fibrils, unlike aducanumab and gantenerumab [25]. Donanemab, on the other hand, has shown high potency against brain A*β* plaques [26]. Although all these drugs decrease A*β* levels in the brain, they have no effect on cognition, except for lecanemab and donanemab [27]. The lack of effect of aducanumab on cognition may be related to limited blood-brain barrier penetration and lack of selectivity for soluble A*β* oligomers. Anti-A*β* monoclonal antibodies can cross the blood-brain barrier (BBB) in several ways, such as modifying brain entry and peripheral sink effects, creating antibody-based carriers, and targeting the BBB receptors. For example, aducanumab has been shown to cross BBB through disruption of BBB, which is also related to ARIA [28]. The transfer of lecanemab and donanemab may also be facilitated by pathological disruption of the BBB induced by AD [29]. In contrast, donanemab transfer across the BBB is facilitated by the use of antibody-based carriers that increase the number of therapeutic proteins that enter the brain through receptor-mediated transcytosis [30]. Lecanemab also uses receptor-mediated transcytosis (RMT) to cross the BBB using bispecific therapeutic antibodies (BSAs). This technique selectively targets BBB receptors such as insulin receptor (IR), leptin receptor (LEPR), transferrin receptor (TfR), and insulin-like growth factor receptor (IGFR) [31]. Bispecific monoclonal antibodies targeting both the transferrin receptor and the A*β* peptide may offer a solution to enhance the delivery of mAbs across the BBB for the treatment of AD. For instance, a tetravalent bispecific antibody that targets transferrin improved the efficacy of disaggregating amyloid plaques in the brains of AD transgenic mice [32].

In a head-to-head comparison study with aducanumab, donanemab outperformed aducanumab in terms of amyloid clearance [33]. A study comparing the incidence of amyloid-related imaging abnormalities (ARIA) among different anti-*β*-amyloid (A*β*) therapies showed that aducanumab is associated with the highest risk of ARIA (ARIA-edema, ARIA-hemorrhage) [34]. Similarly, a higher incidence of ARIA-E was observed with aducanumab than with lecanemab in another study. Donanemab has been associated with a lower incidence of ARIA than aducanumab [33]. The increased risk of ARIA with aducanumab compared to other antiamyloid therapies in AD may be influenced by the drug dose, APOE *ε*4 status, and the unique properties of aducanumab in targeting A*β* aggregates [35]. Due to the lack of head-to-head comparisons and the different study populations enrolled in the respective trials of antiamyloid drugs, direct comparison of the efficacy and safety of these drugs may be limited.

While aducanumab and donanemab require monthly infusions, lecanemab therapy requires infusions every two weeks [32, 33]. Gantenerumab is given as a subcutaneous (SC) injection every two weeks. The duration of therapy with lecanemab is currently indefinite. However, in clinical trials, donanemab is used until the amyloid is cleared [36].

### 1.4. The Catalyst and Imperatives of Alzheimer's Disease Research

Several stakeholders, such as patients, caregivers, national and international associations, and drug companies continue to advocate for AD research. A growing aging population and a better understanding of AD pathogenesis are some of the factors that continue to drive research on AD therapy. The ability to prevent cognitive decline is another crucial gap in the management of AD that fuels research on AD therapy. Another driver for the search for new AD therapies is the lack of effective medication that significantly reverses, stops, or prevents the underlying pathogenesis of AD at the late stages of the disease [37]. In addition, evidence has shown that early intervention is linked to better clinical outcomes in patients with AD. Hence, progress made in developing bioassay tests can enhance the early identification and management of AD [38].

### 1.5. Lecanemab Development and Approval

**Figure 1:**
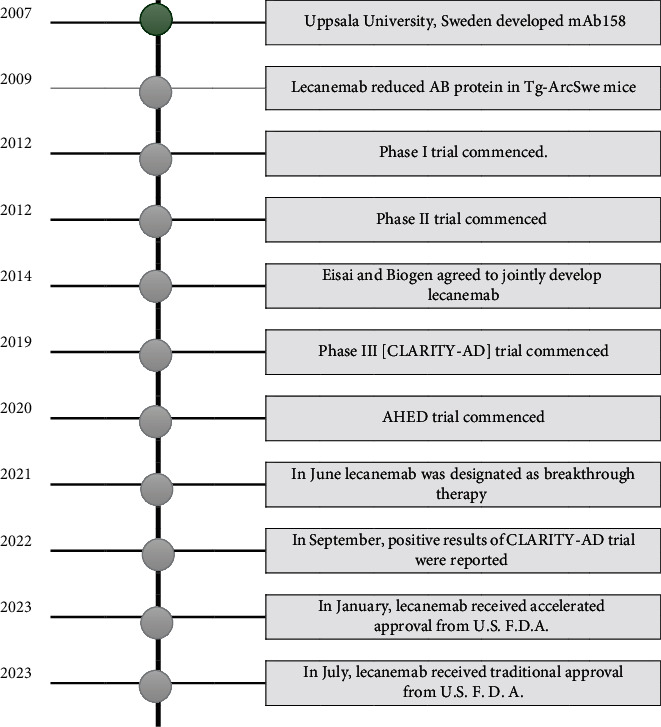
Timeline of lecanemab development, evaluation, and approval. Source: ALZFORUM networking for a cure: therapeutics: Leqembi [101].

Lecanemab is a humanized IgG1 monoclonal antibody that specifically targets the soluble A*β* protofibrils, which are harmful. It removes plaques and prevents the accumulation of these proteins in the brain. Through these actions, lecanemab slows disease progression and cognitive decline.  Figure 1 summarizes the timeline of lecanemab development, evaluation, and approval. The investigational molecule of lecanemab, Mab158, was developed by Uppsala University in Sweden in 2007 [39]. The effectiveness of lecanemab was investigated in preclinical and clinical trials, as shown in Table 1.

After preclinical and clinical studies demonstrated the ability of Mab 158 to reduce A*β* protofibrils in the brain [40], a phase I trial (NCT01230853) was initiated to evaluate the safety, tolerability, and pharmacokinetics of 80 individuals with mild to moderate AD [41]. The drug showed dose-proportional exposure with comparable frequencies of ARIA (ARIA-E, ARIA-H) to placebo [41]. A phase II trial was conducted to determine the most effective dose (ED90) by testing five different doses. This study used a Bayesian adaptive randomization method, favoring doses that would provide more information about ED90 and its effectiveness in identifying the optimal dosage for treating AD. The trial included 854 patients, and because of the randomization method, more individuals were directed to doses that showed superior performance in the clinical trial (NCT01767311) [42]. A total of 609 lecanemab patients and 245 placebo subjects were included in this randomized trial. After a year, with the 10 mg/kg biweekly ED90 dose, the drug failed to reach the main goal. However, at 18 months, using both frequentist and Bayesian methods, the study showed a reduction in brain amyloid levels and a reduction in clinical decline that was consistent across several biomarkers and clinical assessments [43]. The open-label extension (OLE), which included 856 patients on lecanemab 10 mg/kg biweekly, showed sustained treatment benefits over a gap period of 9–59 months, leading to dose-dependent reductions in amyloid PET, improved plasma biomarkers, and maintained clinical differences compared to placebo [44].

The CLARITY-AD phase III trial, which was conducted over 18 months, involved 1795 AD patients with MCI and early AD. Lecanemab, administered as an infusion once every 2 weeks, reduced amyloid in the brain and lowered the clinical dementia rating-sum of boxes (CDR-SB) score by 0.45 points more than the placebo. In addition, the drug was shown to improve the Alzheimer's disease assessment scale-cognitive subscale (ADAS--Cog14), the Alzheimer's disease composite score (ADCOMS), and the Alzheimer's disease cooperative study-activities of daily living scale for mild cognitive impairment (ADCS-MCI-ADL) [45]. The trial demonstrated that lecanemab slows cognitive decline. At 18 months, lecanemab was found to reduce the decline of CDR-SB by 27% compared to placebo. ARIA-E occurred in 12.6% of CLARITY-AD study participants, ARIA-H occurred in 17.3%, and isolated ARIA-H without ARIA-E occurred in 8.9%. However, the trial has been criticized for not adequately representing diverse subgroups of patients with AD. In addition, questions remain about whether the outcomes last over a long period and whether the mild slowing of cognitive decline is clinically significant [46]. Researchers have recommended reporting both the size and variability of outcome measures to assess whether the change is meaningful in addition to the percentage of decline reduction. Moreover, since lecanemab was jointly developed by Eisai and Biogen, potential bias due to the impact of funding on research is anticipated. A simulation study used utility measures of health-related quality of life to assess the impact of lecanemab on the overall health status of patients in the CLARITY-AD trial. The results have shown the potential of this drug to reduce the need for institutional care by slowing disease progression and improving patients' quality of life [47].

The success of the phase II clinical trial and the anticipated positive result of the CLARITY-AD trial (NCT03887455) led to the traditional approval of lecanemab in July 2023. Currently, the AHEAD study (NCT04468659) a phase 3 clinical trial that is aimed at determining whether lecanemab can reduce early stage Alzheimer-related amyloid buildup is underway. This trial included patients aged between 55 and 80 years, categorized based on their amyloid levels, who received lecanemab or a placebo via intravenous infusion for up to 216 weeks. Regular PET brain scans track changes in amyloid and tau levels, providing insights into the impact of drugs [48]. The open label extension (OLE) of the CLARITY-AD trial investigated the weekly SC dose of lecanemab in patients with early AD [49]. This trial enrolled 72 patients with early Alzheimer's who were given SC lecanemab for the first time and 322 patients who were switched from IV lecanemab to SC administration. The AUC of the once-weekly SC lecanemab injection was 11% higher than that of the IV lecanemab infusion once every two weeks lecanemab IV infusion. Injection site reactions were experienced by 15.3% of lecanemab-naïve patients, 8.1% of whom switched from infusion to SC injection. In lecanemab-naïve patients, the incidence of ARIA-E, ARIA-H, and isolated ARIA-H was 16.7%, 22.2%, and 8.3%, respectively [50]. Other studies aimed at assessing the safety and efficacy of lecanemab are currently underway, including NCT05925621, NCT05269394, NCT05999084, NCT01760005, and NCT05469009.

### 1.6. Challenges of Lecanemab Therapy

Lecanemab received traditional approval from the United States Food and Drug Administration (FDA) for the treatment of patients with MCI and mild dementia related to AD in July 2023. However, despite this approval, access to therapy remains unclear for many patients and caregivers, contributing to a delay in access to care. Therefore, healthcare providers are expected to direct patients and caregivers to centers providing lecanemab therapy. A study conducted by the Mayo Clinic highlighted that small proportions of patients with AD can meet the eligibility criteria for receiving aducanumab or lecanemab in clinical practice [51]. Identifying suitable candidates for lecanemab therapy poses challenges for healthcare providers because of various factors. Lecanemab is indicated for early AD associated with MCI and mild dementia, excluding dementia related to the Lewy bodies and Parkinson's disease. Additionally, obstacles, such as limited access to therapy, high costs, inconvenience of drug administration, and difficulties in accessing and interpreting diagnostic tests, particularly in primary care settings, contribute to the complexity of patient selection. Delayed diagnosis and underdiagnosis are particularly common among underserved populations [52]. For this reason, many patients presenting at later stages are less likely to respond to drugs. Diagnostic modalities such as positron emission tomography (PET) and cerebrospinal fluid (CSF) tests are used to confirm the presence of amyloid-beta (A*β*) in the brain [53]. In addition to CT and MRI, plasma biomarkers such as the A*β*42/40 ratio and p-tau181 are currently used in clinical trials to identify patients who require antiamyloid therapy [44]. P-tau is available in the forms of p-tau181, p-tau217, and p-tau23; among these types, p-tau217 is superior in differentiating dementia caused by AD from dementia associated with other diseases [54]. The blood biomarker, phosphorylated tau181, is a strong indicator of amyloid burden. Blood-based biomarkers are now used as an adjunct to PET and CSF evaluations to confirm underlying mild AD and to monitor the effectiveness of antiamyloid medication in clinical trials. Research has shown that these biomarkers are as effective as PET and CSF screening for A*β* protein [55]. However, the use of blood-based biomarkers alone as primary endpoints in AD trials lacks consensus and requires further validation against gold standard measures [56]. Several biomarkers, including plasma-, lipid-, and blood-based biomarkers, are being studied. Plasma assays for A*β* and tau show great promise for clinical and research use [57]. Tests to assess cognitive function are also used in combination with brain scans (CT, MRI, and PET) to identify A*β* or tau accumulation. Tests such as the mini-mental state examination (MMSE) and the Montreal cognitive assessment (MoCA) have modest performance in identifying early stage cognitive decline [58].

In addition, genetic testing is required before the start of lecanemab therapy to avoid administering the drug to patients carrying genetic mutations, such as ApoE *ε*4, which increases the risk of brain hemorrhage or cerebral microbleeds [59] and cerebral amyloid angiopathy (CAA). Patients taking certain medications, such as anticoagulants or antifibrinolytic agents, are advised against lecanemab therapy due to the potential for increased bleeding [60]. Despite the high likelihood of using antidepressant drugs among AD patients on antiamyloid therapy, it is best to avoid starting lecanemab therapy during the first 30 days following selective serotonin reuptake inhibitor (SSRI) administration due to an increased risk of microbleeds [60].

The high cost of lecanemab and the lack of universal insurance coverage pose additional challenges [61]. Collaborative efforts among stakeholders such as patients, clinicians, insurers, manufacturers, and regulatory agencies are crucial in addressing these obstacles and ensuring access to affordable and effective therapies [62]. The cost of lecanemab is estimated to be $26,500 per year, excluding the costs of infusion and monitoring [63]. Currently, companies are developing a maintenance dose with monthly infusion to reduce drug costs. With the recent traditional approval of lecanemab, Centers for Medicare & Medicaid Services (CMS) insurance coverage is warranted only for patients enrolled in a registry [64]. On the other hand, U.S. Veterans Affairs has agreed to cover the costs for veterans with mild AD, except for patients with copies of the ApoE4 gene [65].

Once eligible patients are identified, the requirement for intravenous infusion and regular monitoring may be inconvenient for many patients. Lecanemab requires a twice-monthly intravenous infusion at a dose of 10 mg/kg in patients with early AD and MCI with confirmed A*β* protofibrils. The infusion is administered over 1 h, and patients are required to be observed during and after infusion therapy for infusion-related reactions. The duration of monitoring was approximately 4 h during the first infusion, which could be reduced in subsequent infusions because infusion-related reactions, manifested as fever, chills, headache, nausea, dizziness, or chest tightness, are more common during the first administration. In the CLARITY-AD trial, infusion-related reactions were observed in 20% of the patients on lecanemab. Most infusion-related reactions are mild and self-limiting [45]. Effective administration and monitoring of lecanemab therapy may require specialized infusion centers [66].

In addition to infusion-related reactions, lecanemab increases the risk of ARIA. Therefore, patients who take aducanumab or lecanemab must undergo baseline and periodic MRI to check for ARIA [60, 67]. These adverse effects are typically observed observed during MRI scans. Most cases of ADR are asymptomatic but they can also present with symptoms such as headache, confusion, dizziness, vision changes, nausea, difficulty walking, or seizures [60]. In the CLARITY-AD trial, 21% of individuals receiving lecanemab experienced ARIA [45]. If ARIA is deemed severe, the medication should be discontinued. The decision to restart the medication should be made after assessing the individual patient [60]. Intravenous corticosteroids can be used to treat ARIA symptoms [68]. The MRI sequences used in clinical trials are likely sufficient for effectively detecting cases [69] that may predispose patients to ARIA. Safety concerns have arisen due to two deaths related to lecanemab therapy [42, 64].

### 1.7. Ongoing Trials and Emerging Therapies for Alzheimer's Disease

The Clinical Trials on Alzheimer's Disease (CTAD) Task Force concluded that opportunities for developing effective treatments include the development of new biomarkers, interventions in the early stages of the disease, and the use of combination therapies [70]. There is a growing focus on nonamyloid targets, such as antitau therapies, treatments for inflammation, synaptic and neuronal protection, vascular factors, neurogenesis, and epigenetic interventions. Current research also suggests the need to explore gene and stem cell therapies as effective modalities for treating AD in the future [71].

### 1.8. Antiamyloid Immunotherapeutic Agents

Several studies have investigated the efficacy and safety of immunotherapeutic antibodies that target and remove A*β* plaques. Antiamyloid therapies that target A*β*, such as gantenerumab and donanemab, are being investigated for early AD patients [36]. In a phase 2 trial (NCT03367403) for early AD, the antibody donanemab, which targets a modified form of A*β*, showed promising results. Patients receiving donanemab demonstrated a superior composite score for cognition and daily living activities (iADRS) at 76 weeks compared with those receiving placebo [72]. In the TRAILBLAZER-ALZ 2 trial (NCT04437511), 1,736 amyloid-positive patients with MCI or dementia were randomly assigned to receive either a 72-week placebo or a once-monthly infusion of 1,400 mg donanemab. The cohort was divided into 1,182 individuals with low or medium tau levels and 552 individuals with high tau levels. In patients with low, medium, and high tau pathology, donanemab significantly reduced clinical progression on integrated AD rating scales over 76 weeks compared with placebo in a phase 3 trial for early AD. Donanemab eliminated amyloid plaques in the brain but caused cerebral edema or effusion as side effects in 24% of the treatment group [36]. Donanemab has been shown to have overall clinically significant improvements in the early stages of AD; nevertheless, safety concerns still need to be further investigated [36]. In addition, a post hoc analysis of the TRAILBLAZER-ALZ trial findings revealed that donanemab-induced amyloid reduction at 24 weeks was correlated with baseline amyloid levels. According to modeling predictions, amyloid reaccumulation might take almost 4 years after discontinuing donanemab treatment [73]. An extension study, TRAILBLAZER-EXT (NCT04640077), including those who participated in TRAILBLAZER-ALZ, is recruiting participants to assess the safety and efficacy of the investigational drug donanemab in individuals with Alzheimer's disease and to confirm the validity of video-scale assessments. Unlike donanemab, pooled results from two phase III clinical trials of gantenerumab (GRADUATE I and II) were not associated with a slower clinical decline in patients with early AD [69, 74]. An interventional trial (NCT05552157) that is aimed at ascertaining whether the administration of gantenerumab hinders or decelerates the accumulation of amyloid beta (A*β*) or has an impact on the progression of the disease is underway.

Other immunotherapeutic interventions include AD vaccines and immunogens that are anticipated to stimulate an immune response against various cytotoxic A*β* conformers [75]. Two vaccines, A*β*42 and AOE1, which target the primary amino acid sequence and structural epitope of A*β*, respectively, were studied in EAE/AD mice. These vaccines have demonstrated unique effects on neuropathology and cognitive impairments [76]. BACE inhibitors, which inhibit the production of synaptotoxic A*β*, are also being tested for the treatment of AD. Examples of BACE inhibitors at an advanced stage in clinical development include verubecestat, CNP520, elenbecestat, and lanabecestat. Some trials have shown promising results as disease-modifying agents; however, cognitive-related adverse effects may limit the impact of drugs [72, 73].

### 1.9. Antitau Immunotherapeutic Agents

There has been a shift in focus from antiamyloid therapy to tau-based immunotherapies [77]. Four monoclonal antibodies against tau (gosuranemab, tilavonemab, semorinemab, and zagotenemab) and one antitau vaccine (AADvac1) have been evaluated in phase II clinical trials [78]. Clinical trials of these agents have yielded varied results. For instance, semorinemab demonstrates weak signals of effectiveness in moderate stages of the disease [79], whereas tilavonemab does not show a significant reduction in disease progression [80]. Similarly, gosuranemab did not significantly affect tau buildup or cognitive deterioration in patients with early AD [76, 81]. Theoretically, targeting tau is expected to have a greater impact on cognitive impairment than targeting antiamyloid proteins [82]. In addition, other antitau therapies, such as hydromethylthionine mesylate (HMTM), have shown sustained cognitive improvement in AD [83]. In preclinical testing, salsalate, which prevents tau from being acetylated at Lys174, resulted in reduced p300 HAT activity, which, in turn, led to a decrease in tau acetylation [82].

## 2. Repurposed Drugs for AD Therapy

The field of drug repurposing for AD has grown significantly in the past ten years; however, there is still very little agreement on proposed candidates. Antipsychotics, such as clozapine, aripiprazole, and risperidone, are some of the candidates suggested for repurposing for AD. However, these drugs have not been investigated in clinical trials and are limited by potential side effects in older adults [84]. Existing vaccines against pneumonia and the flu are being considered as other means of AD and dementia prevention [85]. Additionally, another repurposed drug (a tyrosine kinase inhibitor, masitinib) has been shown to reduce cognitive decline in patients with mild-to-moderate AD, as measured by the ADAS-cog14 scale. The drug is tolerable, and the results need to be confirmed through further studies [86].

### 2.1. Gene Therapy for AD

Gene therapy presents a promising treatment option by targeting the underlying biological causes of AD. Gene therapy for AD, which involves changing genes to modify antiamyloid levels, is underway. However, gene therapy for AD is still in its infancy, and further studies are required to evaluate this strategy as a treatment option for AD. Gene therapy is not associated with the side effects linked to systemic protein administration, and sustained protein expression virtually eliminates compliance issues [87]. Numerous delivery vectors—viral and nonviral—are being investigated for effective and safe AD gene therapy. To overcome obstacles in the administration of in vivo gene therapy, nonviral functionalized nanomedicines are being developed. Nanoparticles can be modified to pass the BBB by using different transport channels, such as tight junctions, cellular transport proteins, receptor-mediated transcytosis, transcellular pathways via diffusion, and adsorption-mediated transcytosis [88]. Nanomedicines, such as immunoliposomes or nanoparticles, can be modified to target the transferrin receptor on brain capillary endothelial cells. These nanocarriers can conjugate antibodies or peptides on their surface and interact with the transferrin receptor, triggering transcytosis across the blood-brain barrier (BBB) and release into the brain parenchyma [89]. In a recent study, the use of nanomedicines for brain-targeted gene therapy to treat AD pathology in transgenic mouse models was investigated [90]. While the results of this therapy show promise, its application for AD is limited due to immunogenic reactions and off-target effects [85, 86]. Gene therapies being explored include nerve growth factor (NGF) gene therapy [91], CD33 gene therapy [92], apolipoprotein E (ApoE) 2 gene therapy [93], and lent-glial cell-derived neurotrophic factor (GDNF) gene therapy [94]. Similarly, gene suppression aiming to reduce the expression of genes associated with AD is also being examined [95]. Small interfering RNA (siRNA) can also be used as a potential strategy in treating AD by targeting genes involved in the production of A*β*, a protein associated with the disease [96]. Challenges in delivering the gene to target cells in the brain and the immunogenicity associated with gene therapy remain concerns. Gene therapy has shown limited efficacy in clinical trials, unlike preclinical studies, which show promising results. Some studies suggest that there is limited effectiveness in improving cognitive function in patients. Moreover, gene therapy is often associated with high costs, which can restrict its accessibility for patients. Moreover, the long-term cost-effectiveness of gene therapy for AD is not yet understood.

### 2.2. Cell Therapy for AD

Research on cell therapy for AD is ongoing. Mesenchymal stem cell (MSC) therapy impedes the progression of the disease by promoting neuroregeneration. Additionally, in AD models, extracellular vesicles (EVs) derived from cytokine-preconditioned MSCs have shown immunomodulatory and neuroprotective properties [97]. Stem cell therapy is being explored as a potential treatment for AD. In animal models, therapies involving cell replacement, such as induced pluripotent stem cell-derived neural cells or human embryonic stem cells, have shown promise. However, more research is needed to make these therapies clinically viable [98]. In a recent study, nanoformulation-mediated multifunctional stem cell therapy showed potential for simultaneously removing A*β* and promoting neural regeneration in a murine model [99]. Cell-penetrating peptides (CPPs) with sequences originally derived from a prion protein (PrP) have been shown to exhibit both antiprion and antiamyloid properties, particularly against prion proteins and the A*β* peptide active in AD. These disease-modifying properties have so far been observed in cell cultures and in vitro [100].

This narrative review focused exclusively on studies indexed in Google Scholar, SCOPUS, PubMed, ClinicalTrials.gov, and manufacturers' websites. Despite this limitation, the review offers valuable insights into the rapidly evolving field of AD management. It can serve as a resource for updating medical professionals about the changing landscape of AD therapy and informing those dealing with AD patients about the potential efficacy and drawbacks of new AD therapies.

## 3. Conclusion

Lecanemab is the first antiamyloid drug that has been shown to slow the progression of AD and cognitive decline. It represents a significant step forward in AD management. Its high capacity to remove amyloid protein demonstrates clinical efficacy. However, there is still a gap in treating AD subjects taking anticoagulants or those with 2 copies of ApoE4, severe vascular disease, clotting disorders, preexisting strokes, or seizures. This necessitates more individualized and targeted therapies based on biomarkers and robust monitoring of adverse events. Furthermore, efforts should also be made to increase the representativeness and diversity of clinical trial populations. Additionally, several unexplored pathways remain in AD pathogenesis. This underscores the need to engage AD patients in clinical trials to identify ways to prevent the disease. Combination therapies, with or without lifestyle modification, may be considered. Training healthcare providers, developing policies that improve access to therapy, and promoting public awareness and support for ongoing research efforts in AD are essential.

## Figures and Tables

**Table 1:** Lecanemb clinical trials.

References and trial site	Trial start and completion date	Study design/population	Intervention/comparator	Primary outcome	Results
NCT05925621 A Prospective Comparative Study Of Monoclonal Antibodies For The Treatment Of Alzheimer's Disease Boston, Massachusetts, United States	2023-07-16 to 2028-06	Observational patient registry Prospective comparative study of patients 50 years to 95 years old patientswith a concern for cognitive impairment secondary to AD interested in receiving antiamyloid monoclonal antibody therapy	Lecanemab	To determine whether antiamyloid mabs slow cognitive and functional decline To identify any associations between side effects and patient characteristics To establish the time course of clinical benefits over a period of 30 months	Not available Recruiting

NCT05269394 Dominantly Inherited Alzheimer Network Trial: An Opportunity to Prevent Dementia. A Study of Potential Disease Modifying Treatments in Individuals with a Type of Early Onset Alzheimer's Disease Caused by a Genetic Mutation (DIAN-TU) (DIAN-TU) US, UK, Brazil, Argentina, France, Australia, Canada, Colombia	2021-12-22 to 2027-10	A phase II/III multicenter randomized, double-blind, placebo-controlled platform trial Individuals with a type of early onset AD caused by a specific genetic mutation.	E2814 Lecanemab Matching placebo (E2814)	To assess the change from week 24 to week 104 and week 208 in tau PET in the symptomatic population (cohort 1).	Not available Recruiting

NCT03887455 Study to Confirm Safety and Efficacy of Lecanemab in Participants With Early Alzheimer's Disease (Clarity AD) Australia, Canada, China, France, Germany, Italy, Japan, Korea, Republic of, Russian Federation, Singapore, Spain, Sweden, United Kingdom, United States Swanson et al. [45]	2019-03-27 to 2027-09-15	Phase III interventional study Persons 50 to 90 years of age with early AD (mild cognitive impairment or mild dementia due to AD) with evidence of amyloid on PET or by cerebrospinal fluid testing CSF **Extension phase** Participants reporting one or more treatment-emergent adverse events (TEAEs) **Experimental extension** study will include approximately 40 de novo participants (those that did not participate in the core study) with early AD.	**Core study** Lecanemab 10 mg/kg biweekly Placebo comparator: **Extension phase:** Lecanemab 10 mg/kg biweekly for 51 months Lecanemab 720 mg subcutaneous injection weekly	**Core study:** change from baseline in the CDR-SB at 18 months **Extension phase:** number of participants reporting one or more treatment-emergent adverse events (TEAEs) Change from core study baseline in CDR-SB up to 69 months	Core study included 1795 participants Change at 18 months CDR-SB = −0.45 (−0.67 to − 0.23) Lecanemab = 1.21 Placebo = 1.66 Infusion-related reactions: 26.4% with lecanemab. ARIA with edema or effusions: 12.6%. **Experimental extension** study results not available

NCT05999084 Georgia Memory Net Anti-Amyloid Monoclonal Antibody Registry United States	2023-11 to 2028-07	Observational prospective patient Registry Patients with MCI or mild AD dementia who are receiving treatment with antiamyloid mAbs, per standard of care. Historical comparator group: biomarker-confirmed patients with MCI or mild dementia due to AD who have been followed in the Emory Cognitive Neurology Clinic	Infusions of lecanemab every 2 weeks. Standard of care	To compile information on patients who are receiving FDA-approved antiamyloid mAbs during their clinic visits	Not available Did not start recruiting

NCT04468659 AHEAD 3-45 Study: A Study to Evaluate Efficacy and Safety of Treatment With Lecanemab in Participants With Preclinical Alzheimer's Disease and Elevated Amyloid and Also in Participants With Early Preclinical Alzheimer's Disease and Intermediate Amyloid Australia, Canada, Japan, Netherlands, Singapore, Spain, Sweden, United Kingdom, United States	2020-07-14 to 2027-10-25	Placebo-controlled, double-blind, parallel-treatment arm, 216 week study Male or female, aged 55 to 80 years, with a plasma biomarker result predictive of intermediate or elevated brain amyloid.	Drug: lecanemab Drug: placebo	To determine whether treatment with lecanemab is superior to placebo on change from baseline of the preclinical Alzheimer cognitive composite 5 (PACC5) at 216 weeks of treatment (A45 Trial) and to determine whether treatment with lecanemab is superior to placebo in reducing brain amyloid accumulation as measured by amyloid positron emission tomography (PET) at 216 weeks of treatment (A3 Trial). at Week 216	Not available Recruiting

NCT01767311 A Study to Evaluate Safety, Tolerability, and Efficacy of Lecanemab in Subjects With Early Alzheimer's Disease Canada, France, Germany, Italy, Japan, Korea, Republic of, Netherlands, Spain, Sweden, United Kingdom, United States Swanson et al. [43] McDade et al. [44] Berry, et al. [42]	2012-12-20 to 2025-02-20	A placebo-controlled, double-blind, parallel-group, Bayesian adaptive randomization design and dose regimen-finding study with an open-label extension 856 participants participants will be from 2 clinical subgroups' due to AD or mild AD dementia.	Lecanemab 2.5 mg/kg Lecanemab 5.0 mg/kg Lecanemab 10 mg/kg Lecanemab 5.0 mg/kg Lecanemab 10 mg/kg Drug: placebo Lecanemab 10 mg/kg	**Core Study:** change from baseline in the AD composite score (ADCOMS) at 12 months. **Core study and extension phase**: safety will be assessed by monitoring and recording all adverse events (AEs) and serious adverse events (SAEs)	12 months: 10 mg/kg biweekly dose of lecanemab (ED90) had a 64% probability of being better than placebo by 25% on ADCOMS. 18 months: at 10 mg/kg biweekly, lecanemab reduced brain amyloid by -0.306 SUVr units. Drug-placebo differences favored active treatment: ADCOMS: 27% (Bayesian analysis), 30% (frequentist analysis). ADAS-Cog14: 56% (Bayesian), 47% (frequentist). CDR-SB: 33% (Bayesian), 26% (frequentist). CSF biomarkers: lecanemab was well-tolerated. The incidence of ARIA-edema/effusion at 10 mg/kg biweekly was 9.9% lecanemab 10 mg/kg biweekly treatment in the OLE demonstrated dose-dependent reductions in amyloid PET SUVr and improvement in plasma A*β*42/40 ratio and reduction in plasma p-tau181 levels.

NCT01760005 Dominantly Inherited Alzheimer Network Trial: An Opportunity to Prevent Dementia. A Study of Potential Disease Modifying Treatments in Individuals at Risk for or With a Type of Early Onset Alzheimer's Disease Caused by a Genetic Mutation. Master Protocol DIAN-TU-001 (DIAN-TU) Argentina, Australia, Brazil, Canada, Colombia, France, Germany, Ireland, Italy, Japan, Mexico, Netherlands, Puerto Rico, Spain, United Kingdom, United States	2012-12 to 2027-10	A phase II/III multicenter randomized, double-blind, placebo-controlled platform trial Participants with an AD-causing mutation	Gantenerumab solanezumab Matching placebo (gantenerumab) Matching placebo (solanezumab) E2814 Lecanemab Matching placebo (E2814)	To assess the safety, tolerability, biomarker, cognitive and clinical efficacy of investigational products (e.g., slowing the rate of progression of cognitive/clinical impairment or improvement in disease-related biomarkers.	Recruiting Not available

NCT05469009 Safety and Feasibility of Exablate Blood-Brain Barrier Disruption for Mild Cognitive Impairment or Mild Alzheimer's Disease Undergoing Standard of Care Monoclonal Antibody (mAb) Therapy United States	2022-07-14 to 2029-07	Interventional early phase I study Patients with MCI or mild AD	Aducanumab Device: Exablate Model 4000 Type 2 Lecanemab	Treatment intervention-related adverse events, total number of adverse events following each treatment, treatment intervention-related serious adverse events, and the total number of serious adverse events following each treatment	Recruiting Not available

NCT01230853 A Randomized, Double-blind, Placebo-controlled, Combined Single Ascending Dose and Multiple Ascending Dose Study Logovinsky et al. [41] United States	2010-08 2012-10	A randomized, bouble-blind, placebo-controlled, combined single ascending dose and multiple ascending dose study The population under investigation consists of individuals with mild to moderate AD.	Drug: active comparator: A Drug: placebo comparator B Drug: active comparator B Drug: placebo comparator A	The safety and tolerability of BAN2401. ARIA on MRI, specifically assessing edema (ARIA-E) and hemorrhage (ARIA-H), were monitored. Pharmacokinetics PK of BAN2401 in CSF and plasma samples were analyzed. To study the effect of the drug on CSF biomarkers	The incidence of ARIA-E/H with BAN2401 was comparable to the placebo group. BAN2401 exposure demonstrated dose proportionality. Serum terminal elimination half-life: the serum terminal elimination half-life of BAN2401 was approximately 7 days. A slight increase in plasma A*β* (1-40) was observed with BAN2401. CSF Biomarkers: no measurable effects of BAN2401 on CSF biomarkers were detected. Phase 2b Efficacy study is initiated

Abbreviations: AD: Alzheimer's disease; ADC: Alzheimer's disease composite; ADAS-cog14: Alzheimer's disease assessment scale-cognitive subscale (14 items); ADCI: Alzheimer's disease composite score; ADCS: Alzheimer's disease cooperative study; ARIA: amyloid-related imaging abnormalities; CDR-SB: clinical dementia rating-sum of boxes; CSF: cerebrospinal fluid; DIAN-TU: dominantly inherited Alzheimer network trial: an opportunity to prevent dementia; FDA: U.S. Food and Drug Administration; MCI: mild cognitive impairment; PET: positron emission tomography; TEAE: treatment-emergent adverse event.

## Data Availability

The datasets used during the current study are available from the corresponding author upon reasonable request.
